# Reconsidering Visual Search

**DOI:** 10.1177/2041669515614670

**Published:** 2015-11-08

**Authors:** Árni Kristjánsson

**Affiliations:** Department of Psychology, School of Health Sciences, University of Iceland, Iceland

**Keywords:** Visual attention, visual search, slopes, serial models, parallel models

## Abstract

The visual search paradigm has had an enormous impact in many fields. A theme running through this literature has been the distinction between preattentive and attentive processing, which I refer to as the *two-stage* assumption. Under this assumption, slopes of set-size and response time are used to determine whether attention is needed for a given task or not. Even though a lot of findings question this two-stage assumption, it still has enormous influence, determining decisions on whether papers are published or research funded. The results described here show that the two-stage assumption leads to very different conclusions about the operation of attention for *identical* search tasks based only on changes in response (presence/absence versus Go/No-go responses). Slopes are therefore an ambiguous measure of attentional involvement. Overall, the results suggest that the two-stage model cannot explain all findings on visual search, and they highlight how slopes of response time and set-size should only be used with caution.

## Introduction

The well-known visual search paradigm has had an enormous impact on many aspects of science. The paradigm was designed to assess the function of visual attention (Neisser, 1963; Treisman & Gelade, 1980; Wolfe, 1998) and the operation of early visual cortical areas (Julesz, 1981; Nakayama & Martini, 2011). But its’ subsequent reach has been far wider than this, and key assumptions about attention derived from an outdated understanding of the paradigm are used as blueprints for inference in many disciplines.

In the vast majority of visual search studies, performance is assessed with response times. Typically, the task is to determine whether a target is present or absent. Search slopes that measure how response times change as more distractors are added to the display are presumed to assess the speed of the search and whether attention is involved. If the slopes are around zero, the common assumption is that the target can be detected *preattentively*, while if search times increase with set-size, this is considered to reflect that *attention* moves around the search array, seeking the target. Even if an overall change in mean is observed, it is not thought to reflect the operation of attention if slopes are constant. I will refer to this preattentive or attentive distinction as the *two-stage assumption*.

A common finding is that when a target differs from distractors on a single feature search slopes are around 0, reflecting that search is unaffected by the number of distractors in a display. *Conjunction* searches where the target shares features with each distractor type often result in inefficient search where response times (RT’s) increase by 10 to 50 ms with each added distractor item (Wolfe, 1998a).

While this logic is in many ways attractive, note importantly that it is a *testable assumption* about visual attention (Duncan & Humphreys, 1989; Treisman & Gelade, 1980; Wolfe, Cave, & Franzel, 1989). Other models of visual search have been proposed that do not involve the two-stage assumption, such as parallel models (Eckstein, 1998; Palmer, 1995), serial exhaustive models (Sternberg, 1975), and models that postulate a race for recognition by parallel processing of bottom–up and top–down information (Bundesen & Habekost, 2008). Approaches that assume that observers adapt to the search environment, such as with Bayesian updating of preferences and biases (e.g. Vincent, 2015) also show promise in accounting for the operation of visual attention. All these approaches bypass the serial or parallel distinction (see also Wolfe, 2007).

### The Problem

The standard two-stage assumption of visual search is often used to infer facts about visual attention but many researchers overlook that it is exactly that—an assumption. Slopes of response times versus set-size are used to *decide* whether visual attention is involved or not: If a particular manipulation affects slopes, this is thought to reflect attentional processing, but unchanged slopes reflect that the manipulation does not affect attention. Note that this is often taken beyond reason through circular reasoning: If search slopes for a given task are around 0, the search is considered “preattentive.” To determine what is analysed preattentively, the tasks that lead to flat search slopes simply need to be identified. There may, of course, be other reasons for the flat search slopes.

A few random examples from diverse fields of how the two-stage assumption is used as fact rather than conjecture are mentioned here (almost literally picked out of a hat from among many possible ones). All highlight the contaminating influence of the two-stage assumption. In visual masking, Argyropoulos, Gellatly, Pilling, and Carter (2013; see Filmer, Mattingley, & Dux, 2014 for similar assumptions) concluded that object substitution masking was not obliterated by attention as originally claimed by Enns and DiLollo (1997), since they found that such masking was not affected by set-size manipulations. There was little discussion of the reasoning behind the assumption that set-size effects are indicative of attentional effects. This was simply taken as given. Another example involves research into the relation of working memory and attention. Woodman, Vogel, and Luck (2001) investigated interactions of working memory and attention, finding that added working memory load did not influence set-size effects in visual search. This led them to conclude that visual search requires minimal working memory resources, inconsistent with ideas of overlap in function of attention and working memory (e.g. Awh & Jonides, 2001; Bundesen, 1990). Notably, they found a large intercept effect but stated that: “this change in intercept implies that the memory task led to a slowing of a process that either preceded or followed the actual search” (p. 221), again a conclusion based on the two-stage assumption. Oh and Kim (2004) subsequently argued that there was dual-task interference in a task involving spatial visual memory and visual search. Again set-size effects were the key to reaching this conclusion. Horowitz and Wolfe (1998) investigated the role of spatial memory in visual search finding that randomly relocating search items every 110 ms during search did not modulate set-size effects. They concluded that attention did not keep track of already searched locations. They did, however, find intercept effects from the reshuffling. Kristjánsson (2000) questioned their conclusion by modifying their paradigm, but again the reasoning depended on the slopes, although Kristjánsson noted that intercept differences could have relevance for attention under other models than those involving the two-stage assumption. Greenberg et al. (2015) investigated object-based attention arguing that the attentional priority surrounding a selected object is modulated by search mode as measured with slopes.

The two-stage assumption is applied to research outside the field of visual cognition. In clinical psychology, Hahn, Carlson, Singer, and Gronlund (2006) used slopes of response time and set-size to assess attentional bias to threatening faces. In neuropsychology, Ashwin, Wheelwright, and Baron-Cohen (2006) investigated the anger superiority effect in face perception, using slopes to measure attention to facial expression in Asperger syndrome. In a study of neglect patients, the current author is again guilty. Kristjánsson and Vuilleumier (2010) used search slopes to argue for a difference in spatial memory between visual hemi fields, but to their credit they noted caveats regarding assumptions about attentional involvement from slopes. In the controversial field of action video-game training (see Boot, Blakely, & Simons, 2011; Kristjánsson, 2013 for critical overviews), Hubert-Wallender, Green, Sugarman, and Bavelier (2011) stated that search slopes could be used as a diagnostic tool for measuring influences on attentional processing from video-game training or experience (see also Castel, Pratt, & Drummond, 2005; Wu & Spence, 2013). In Neurophysiology, the operative characteristics of neurons in various vision and attention-related brain regions have been assessed under the two-stage assumption (Cohen, Heitz, Woodman, & Schall. 2009; Jerde, Ikkai, & Curtis, 2011; see also Chelazzi, 1999). Similar assumptions have been made in neuroimaging research on humans (Donner et al., 2002; Nobre, Coull, Walsh, & Frith, 2003) . All these studies take the two-stage model as given. Finally, the two-stage assumption is found in the Wikipedia entry on visual search, not stated as conjecture, but fact (http://en.wikipedia.org/wiki/Visual_search#Reaction_time_slope, retrieved May 1^st^, 2015). In sum, the original theoretical claims that were based on a particular interpretation of visual search data have been used at face-value as facts about attention, not testable hypotheses.

### Findings question the two-stage assumption

It would of course be fine to accept and apply the two-stage assumption had it stood the test of time. It did, however, not take long for results to appear that did not fit with the two-stage formulation. Examples are efficient conjunction search (e.g. McLeod, Driver, & Crisp, 1988; Nakayama & Silverman, 1986; Theeuwes & Kooi, 1994; Wolfe et al., 1989) and set-size effects where response times *decreased* with larger set-size (Bravo & Nakayama, 1992; see also Bacon & Egeth, 1991; Santhi & Reeves, 2004 (Experiment 3); Schoonveld, Shimozaki, & Eckstein, 2007) showing how target salience can *increase* with more nontargets. The same features have been found to function very differently depending on context: The spatial layout of *identical* features can determine whether search is efficient or not (Enns & Rensink, 1990), and familiarity strongly affects visual search for the same basic features (Wang, Cavanagh, & Green, 1994). In the aforementioned study of Horowitz and Wolfe (1998), where search items were reshuffled around the scene every 110 ms, whether there was in fact a deficit in search slopes may be irrelevant to the deeper point that the results (and those of Kristjánsson, 2000) were quite inconsistent with the two-stage assumption. The reshuffling effects were simply far too small for this, regardless of whether memory was involved or not. Joseph, Chun, and Nakayama (1997) found that an attention-demanding task presented at center interfered with presumed preattentive search. Other examples of results that strongly contradict the two-stage assumption are findings on foraging (Kristjánsson, Jóhannesson, & Thornton, 2014), analyses of response time distributions (Palmer, Horowitz, Torralba, & Wolfe, 2011, Kristjánsson & Jóhannesson, 2014; Wolfe, Palmer, & Horowitz, 2010), and of the distribution of search slopes as a function of task (Wolfe, 1998). Even though leading theories of attention have responded to these findings (e.g. Wolfe 1994, 2007), the simple version of the two-stage assumption is still applied to research in diverse areas, as the examples discussed above show.

Wang, Kristjánsson, and Nakayama (2005) investigated a “multiconjunction” search task where the target could neither be distinguished from distractor based on single features nor on the basis of top–down feature biases. In a typical example, the target was randomly either a black disk or a white donut, while the distractors were black donuts and white disks. The two target types share a feature with both distractor types minimising target saliency. According to the two-stage assumption, the visual system would treat target and distractors alike and need to search through the display items one by one, while the only useful top–down guidance signal is that the target is always the odd-one-out. Many visual search theories (Duncan & Humphreys, 1989; Treisman & Sato, 1990; Wolfe et al., 1989) clearly predict inefficient search on such tasks since the primary determinants of visual search performance are, by these accounts, feature contrasts (preattentive) and top–down biases toward certain features (attentive). Yet set-size effects were small, or nonexistent in many examples, indicating that the search was “efficient” (Wolfe, 1998a).

### Go/No-go measures

There are other ways of assessing visual search performance than response times on a present or absent (PA) task. Another finding in the aforementioned paper of Wang et al. (2005, Experiment 4) is less well known. When they changed their multiconjunction search task, so that observers did not have to respond whether the target was present or absent, but only if the target was present (and do nothing if no target was absent; a “Go/No-go” task, GNG), search slopes in multiconjunction search actually became negative—search times *decreased* with set-size. Even if the task was switched so that observers only responded on trials with no target, where search slopes should be twice the size of target present slopes under the two-stage assumption, the slopes were close to zero. Additionally, the intercepts were much lower (by ∼50%) than for a PA task with *identical* search stimuli. Chmiel (1989) found that target present slopes for conjunction search became flat when the search task changed from involving a PA decision to a GNG decision as did van der Heijden and La Heij (1982) who also observed the error rates were lower for the GNG task.

The important point to take from this is that search performance can be very different for the *same* search task with only slight modification of response. But, more seriously, this means that under the two-stage assumption, visual attention operates differently for *identical* searches depending on response requirements. Wang et al. (2005) raised another point worth considering. There may be a task-discrepancy between the present and absent decisions in typical visual search that gives the target-presence decision precedence over the absent decision. Only when the observer fails to find a target is the absent decision an option.

Additionally, Chun & Wolfe (1996) suggested that participants tend to be less certain when they do not see a target and thus examine more of the display before making a decision. Wang et al. did indeed observe that error rates did *not* differ for present and absent trials for the GNG task. Error rates were, in fact, much lower overall for the GNG than the PA task (see also van der Heijden & La Heij, 1982). This may be because the GNG task allows the decision to involve *detecting* target presence versus absence. Making the absent or present decisions the primary task under different conditions may therefore be a way of equating the tasks.

### Current experiments

**Figure 1.**
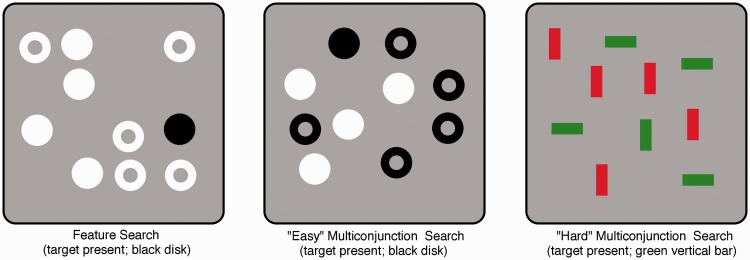
The three visual search tasks that were tested, both with traditional Present/Absent (PA) responses or Go/No-Go/(GNG) responses (all shown with set-size = 10). Feature search on the left, where a single feature (brightness) distinguishes target and distractors; “easy” multiconjunction search at center where the target shares one feature with each distractor set and “hard” multiconjunction search on the right. A target is present in all the examples. The target was randomly one of the four possible search items in each search on a given trial.

Here the GNG visual search paradigm used by Wang et al. (2005) is revisited (along with the more traditional PA task) to assess whether the two-stage assumption leads to similar conclusions about attention from GNG and PA results. To get a relatively wide range of estimates of PA versus GNG performance, three different tasks were tested (see Figure 1):

1. Search based on a single feature with four possible targets (black disk, black donut, white disk, or white donut). Both distractor sets always shared a feature that the target did not have, so the target was distinguished from distractors on a single feature. For example, for a black disk target, the two distractor sets were either white disks and white donuts or white and black donuts.
2. “Easy” multiconjunction search with four possible targets (black disk, black donut, white disk, or white donut). Either distractor set shared one feature with the target, but importantly, never the same one so that no single feature ever distinguished target from distractors. So if the target was a black disk, the distractors would be white disks and black donuts.
3. “Hard” multiconjunction search with four possible targets (red vertical or horizontal bar, or green vertical or horizontal bar). Again, either distractor set shared one feature with the target, so that no single feature ever distinguished target and distractors. If the target was, say, a red vertical bar, the distractors would be red horizontal and green vertical bars.

## Methods

*Observers.* Eight observers (six male, mean age 31) participated. All had normal, or corrected to normal vision. One additional participant was excluded because of incomplete data. They participated in 200 trials under each of nine conditions (The three response conditions: PA, GNG—present, GNG—absent, tested for the three different search task), 1,800 trials in total.

*Equipment.* The experimental displays were programmed in C using the VisionShell software library and presented on an 85-Hz CRT controlled by a 400-MHz G4 Apple computer.

*Stimuli and procedure*. Each trial in both tasks started with the presentation of a central white (56.6 cdm^–2^) fixation cross. Following a variable interval (1,000–1,500 ms, randomly determined for each trial), the experimental stimuli appeared. Auditory feedback was provided on whether the answer was correct or incorrect. The viewing distance was 60 cm. The search items appeared on an approximately midgray background (33 cd m^–2^). In all conditions, set-size was 10, 18, 24, or 32 items determined randomly for each trial. The search items were equally distributed on an invisible 8 × 8 grid (cell size = 2.2°) with a slight random position jitter (±0.4°) within each cell to introduce irregularity. The task was to determine whether an odd-one-out target was present or not (PA or GNG response, see below).

*Feature search task.* The four possible search items were black (0.8 cdm^–2^) or white (56.6 cdm^–2^) annuli or black or white disks (see Figure 1, left). The target always differed from distractors by having a *unique* feature. The disks and donuts had a diameter of 1.1°.

*Multiconjunction search task with disks and donuts.* The target always *shared* a feature with each of the two distractor types (e.g. a white donut among white disks and black donuts; see Figure 1b center).

*Multiconjunction search task with bars of varied color and orientation*. The target always shared a feature with each of the two distractor types (e.g. a red (22.7 cdm^–2^) vertical bar (size 1.2° by 0.4°) among green (27.6 cdm^–2^) vertical and red horizontal bars (Figure 1).

There were three different response conditions run in counterbalanced order (two 100 trial blocks for each condition). In a GNG-presence session, observers responded with a keypress only if the target was present (on 50% of trials) but were told to “sit and wait” if the target was absent, in which case the trial ended after 2000–3500 ms (randomly determined). In a GNG-absence session, observers responded only if the target was absent. In the PA task, observers determined whether the target was present or absent by pressing corresponding keys.

## Results and Discussion

**Figure 2.**
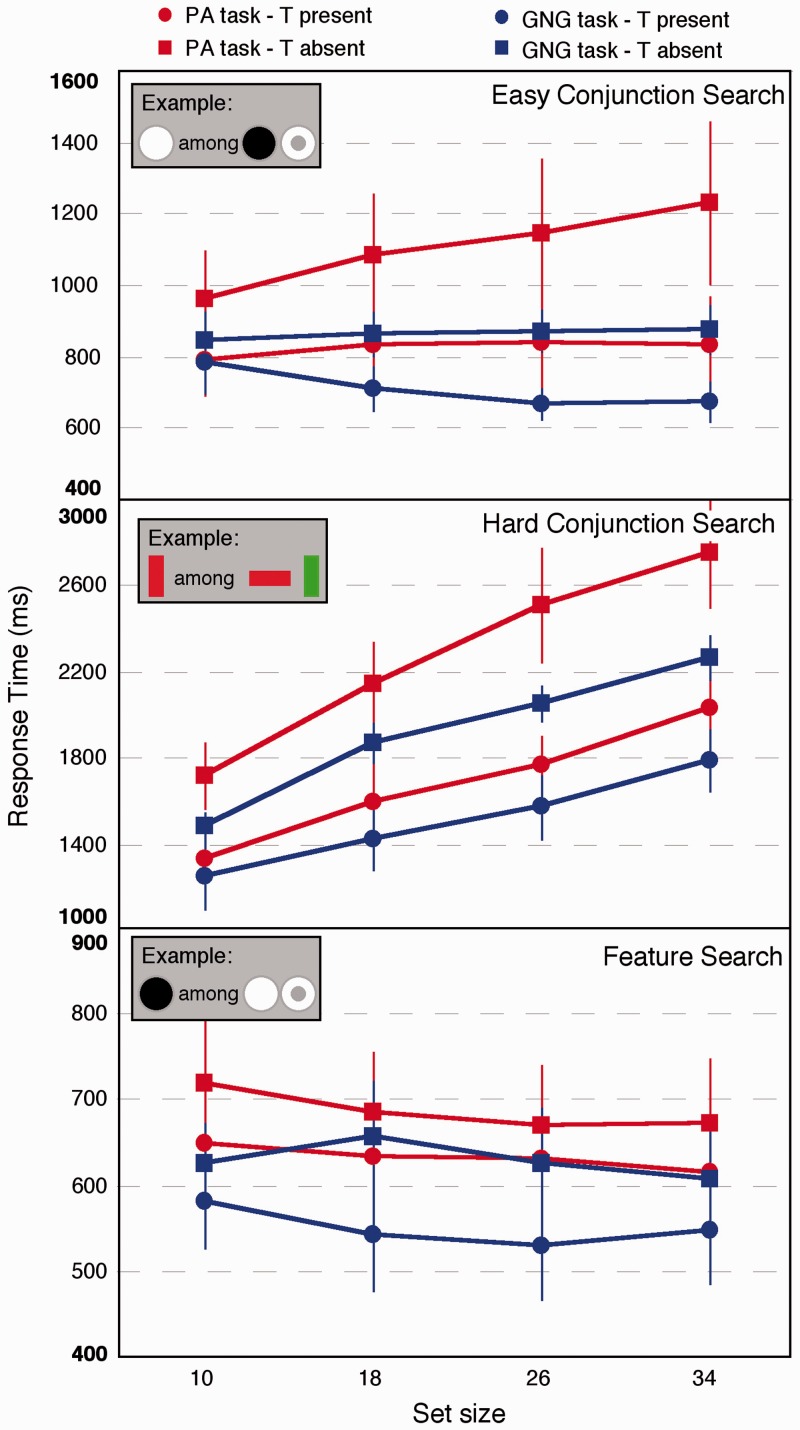
The response times for the present/absent versus Go/No-go tasks for the three different searches. Note the difference in scales, which reflect the differences in overall means for the different conditions. Error bars show the standard errors of the mean.

*(1) Easy Conjunction Search With Discs and Donuts.* The response times from the easy conjunction task are shown in Figure 2 (top). A number of findings should be highlighted. First, the set-size effect differs by task (slopes and intercepts are presented in Table 1). When the target is present, the slopes are *negative* in the GNG task but positive for the PA task. When the target is absent, the RTs are almost 5 ms larger per added item in the PA task than the GNG task.

**Table 1.** Slopes and Intercepts for the Different Search Types and Response Conditions (in ms).

	Easy conjunction Search		Hard conjunction Search		Feature search	
	Intercept	Slope	Intercept	Slope	Intercept	Slope
PA—present	765	2	1,058	23	685	–2
PA—absent	867	11	1,249	48	736	–2
GNG—present	806	–4	1,059	17	592	–1
GNG—absent	798	6	1,318	27	653	–1

*Note.* PA = Present/Absent; GNG = Go/No-Go.

**Figure 3.**
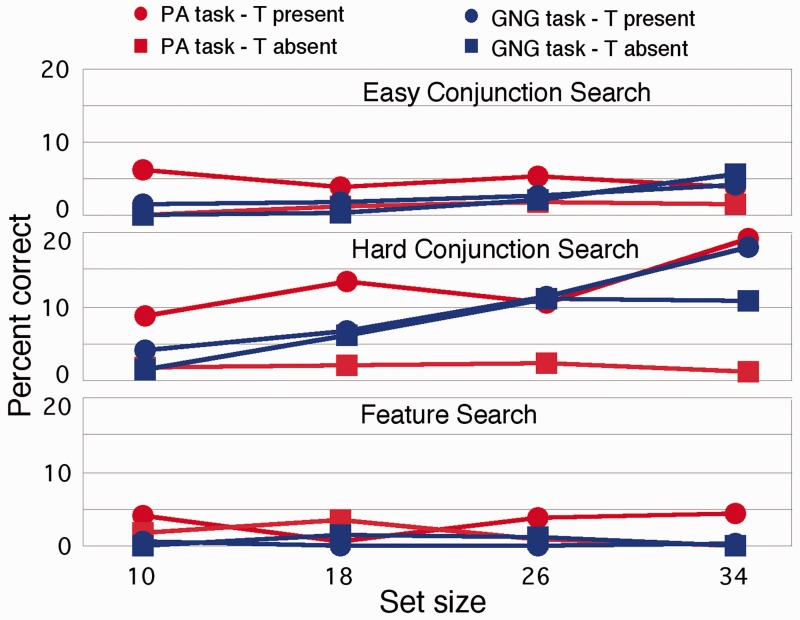
Error rates for the present/absent versus Go/No-go tasks for the three different searches.

What this means is that under the two-stage assumption, we are forced to very different conclusions about the attentional requirements of the task. According to standard theory, this is a conjunction search where the target does not pop-out nor can top-down feature guidance be applied since the target is not known. The fact that the slopes are very small in the PA multiconjunction search is troubling for the two-stage assumption on its own since the results suggest that the search is “preattentive.” But the results from the GNG task are even harder to account for under the two-stage assumption. Attention seems to be doing something quite different from what the two-stage assumption dictates. Attention is clearly not moving from item to item trying to locate the target. Instead, observers are making a decision that becomes *easier* as more items are added to the display. More generally, this means that slopes are an ambiguous measure of visual attention. A three-way analysis of variance (ANOVA; task, set-size, and target presence versus absence) showed that the main effect of task was not quite significant (*F*(1,7) = 3.643; 0.0979; partial *η^2^*^ ^= .34), a significant effect of set-size (*F*(3,21) = 3.883; *p* = .0236; partial *η^2^*^ ^= .33), and a significant effect of PA (*F*(1,7) = 22.45; *p* = .00211; partial *η^2^*^ ^= .76). Importantly, there was an interaction of set-size and task, showing how the slopes of RT and set-size differ (*F*(3,21) = 3.338; *p* = .0389; partial *η^2^*^ ^= .34) by response. Additionally, there was a large interaction of set-size and presence (*F*(3,21) = 13.74; *p* < .001; partial *η^2^*^ ^= .2). Other interactions were not significant. The error rates (presented in Figure 3) were very low. Error rates would have to be far larger and differential by condition for strategy effects, such as speed or accuracy trade-offs to account for the results.

(2) *Hard Conjunction Search With Colored Oriented Bars.* The results for the hard conjunction search are shown in Figure 1 (middle, slopes and intercepts in Table 1). Again, we are forced to quite different conclusions regarding attention by a slight modification of the response. There are both large differences in slope and intercept between the PA and GNG conditions. A three-way ANOVA revealed a large effect of task (*F*(1,7) = 16.91; *p* = .005; partial *η^2^*^ ^= .71), of set-size (*F*(3,21) = 118.1; *p* < .0012.62; partial *η^2^*^ ^= .31), and of target presence (*F*(1,7) = 162.7; *p* < .001; partial *η^2^*^ ^= .96). There was an interaction of task and set-size, showing again that the slopes differ by task (*F*(3,21) = 3.579; *p* = .031; partial *η^2^*^ ^= .11). Finally, the commonly seen interaction between target presence and set-size was significant (*F*(3,21) = 10.53; *p* = .001; partial *η^2^*^ ^= .2). Again, the two-stage assumption yields a very different conclusion as a function of task since slopes are the measure of the speed of attentional processing under that account. The error rates (Figure 3) suggest that speed or accuracy trade-offs are unlikely to account for the results. They are highest for the target present case in the PA condition. Performance under this condition may therefore be *over*estimated, which would mean that the results *underestimate* the difference between GNG and PA performance. Note, however, that there may be a slight difference in slopes between the PA and GNG tasks in the error rates, which may reflect some differences in speed–accuracy trade-offs. This evidence is not strong, however, and is unlikely to account for the slope differences seen for the RT data.

*(3) Feature Search.* The results for the feature search are shown in Figure 1 (bottom; slopes and intercepts shown in Table 1). Immediately notable is that the response times time *decrease* overall with increased set-size. Furthermore, performance is faster for the GNG task revealing large intercept effects in a feature search task that are solely due to changes in response method. This difference might reflect changes in strategy during the visual task itself, since in the GNG task, observers must *detect* presence or *detect* absence, rather than absence being a decision made only when no target is found. A three-way ANOVA revealed that the main effect of task (PA vs GNG) was not quite significant (*F*(1,7) = 3.13 by *p* = .12; partial *η^2^*^ ^= .31). The main effect of set-size was significant (*F*(3,21) = 6.48, *p* = .003; partial *η^2^*^ ^= .16), confirming decreased RTs with increased set-size, as was the effect of target presence versus absence (*F*(1,7) = 9.14; *p* = .019; partial *η^2^*^ ^= .57). No interactions were significant. Error rates (Figure 3) were very low and would have to be far larger and differential by condition for strategy effects, such as speed or accuracy trade-offs to account for the results.

## General Discussion

The results clearly show how slopes are ambiguous measures of visual attention. Three very different patterns of results are seen for the three different search tasks, but importantly, the slopes differ as a function of response method. The idea that slopes measure attention derives from the assumption of a preattentive stage followed by an attentive stage where the search is carried out. This *two-stage* assumption therefore leads to differing conclusions about attentional function from the current results—different conclusions that are based solely on slight modification of response method. This result shows how problematic strict adherence to the two-stage assumption can be. The two-stage model and corresponding slopes should only be used with great caution, and probably should not be used to determine whether attention is involved in a particular task or not.

It may surprise some readers to see this argument made in 2015. Some may feel that a straw man has been erected. Others may wonder: “who cares?” Findings that the two-stage assumption cannot account for have been around in the literature since the 1980s (see examples in Introduction section). But keep in mind that the implicit influence of the two-stage assumption is still strong as examples described in the introduction show. Interpretation of research findings is in many cases based on the two-stage assumption meaning that scientist may draw incorrect conclusions from their results. Arguments made in many disciplines in recently published papers depend on the assumption that if you do not modulate slopes of set-size and RT you do not measure attention. What is important regarding the current results, above other counterexamples to the two-stage assumption is that the differing conclusions are obtained on tasks requiring *identical* searches where only response instructions are changed. Under the two-stage assumption, such instruction variability should not affect processes connected with the search. Data on the *same search* leave us with different assumptions about attention.

### What’s going on during visual search

Multiconjunction search, results from foraging tasks, analyses of RT distributions or the distribution of search slopes, along with the findings described in the introduction, argue against the two-stage assumption. This highlights that the field still has not figured out how visual search works. Slopes are clearly not a straightforward measure of attention as has been the dominant view for 35 years.

What are we left with? Rauschenberger (2010, p. 110) questioned the bottom–up or top–down distinction that is central to the two-stage view, stating: “It is entirely conceivable that there is no temporally or spatially localisable final output that represents the final word in how the brain interprets a given visual input” and later: “In this scheme, “top–down” and “bottom–up” make little sense because there is no isolated feed-forward volley.” I agree with Rauschenberger; it may be time to stop thinking linearly about vision and attention. Rauschenberger points toward the importance of reentrant processing (Ahissar & Hochstein, 2004; Lamme & Roelfsema, 2000); and feedback connections. These proposals strongly blur any distinction between “bottom–up” versus “top–down” processing.

Nakayama & Martini (2011) suggested that visual search might be considered a pattern recognition task. Eckstein (1998) suggested that differences between feature and conjunction search reflected noisy interactions among different features. Such accounts might, for example, explain negative slopes, since with more search items there may be more evidence to base a decision on. Related to this, Wang et al. (2005) argued that perceptual organisation could play a large role in search; organisation that might be *easier* with increased set-size. But these speculations may at best help explain the negative slopes seen here, and at this point, no concrete theory accounts for all visual search findings.

Visual search may very well be so multifaceted that no single idea will explain all the data. Nakayama & Martini (2011) discuss many similar points to those that I make, stating that a new conception of visual search is still “[…] in embryonic form, but discernible is a core which de-emphasizes canonical detectors, ignores the ‘binding’ problems and allows for very high level processing to occur very early in time” (p. 109). In some ways this is akin to the proposals of Julesz (1981) and his *texton* theory, although Julesz distinguished between preattentive and attentive processing stages, which may not fit well with the current zeitgeist (Awh, Belopolsky, & Theeuwes, 2012; Kristjánsson, 2010; Rauschenberger, 2010). Alternative models, such as those discussed in the introduction where the two-stage dichotomy is ignored, may be better fits to this conception. Parallel models have, for example, been successful in accounting for decreases in RT with increased set-size (Santhi & Reeves, 2004; Schoonveld et al., 2007).

### Conclusions

Most researchers have abandoned strong versions of the two-stage model of visual search and attention. Nevertheless, it still exerts a strong influence upon many fields in Psychology and Cognitive Neuroscience. My data argue against this model, and so do many other findings. Clearly, it should only be used with great caution.
